# DOK2 Has Prognostic and Immunologic Significance in Adults With Acute Myeloid Leukemia: A Novel Immune-Related Therapeutic Target

**DOI:** 10.3389/fmed.2022.842383

**Published:** 2022-03-07

**Authors:** Jiaxuan Xu, Xiaoqing Dong, Ruoyi Wang, Bing Chen

**Affiliations:** Department of Hematology, Affiliated Drum Tower Hospital, Medical School of Nanjing University, Nanjing, China

**Keywords:** DOK2, prognostic biomarker, acute myeloid leukemia, immune microenvironment, therapeutic target

## Abstract

**Background:**

The role of downstream tyrosine kinase 2 (DOK2), a major member of the DOK family, remains poorly defined in acute myeloid leukemia (AML). Herein, we investigated the expression levels, clinical outcomes, and biological functions of DOK2 in patients with AML.

**Methods:**

Datasets were obtained from the Cancer Genome Atlas (TCGA) database for transcriptomic and clinical information. Nomogram construction and assessment were conducted using Cox regression analysis, receiver operating characteristic (ROC) curves, and calibration plots. Public databases, including the Gene Expression Omnibus, Cancer Cell Line Encyclopedia, LinkedOmics, GeneMANIA, TISIDB, and Gene Set Cancer Analysis, were employed for relevant bioinformatic studies. Moreover, we utilized the CIBERSORT algorithm to evaluate the level of infiltration of immune cells into the bone marrow microenvironment.

**Results:**

We observed that DOK2 transcription levels were markedly upregulated in AML samples (*P* < 0.001), and its high expression was associated with inferior overall survival (OS) (HR = 2.17, *P* < 0.001) and disease-free survival (DFS) (HR = 2.50, *P* < 0.001). ROC curve analysis also showed the reliable diagnostic efficiency of DOK2 in AML. For treatment regimens, patients with high DOK2 expression could significantly prolong OS by receiving hematopoietic stem cell transplantation (HSCT) (*P* < 0.001), whereas those with low DOK2 expression were more likely to improve DFS by chemotherapy alone rather than HSCT. Nomograms constructed for predicting OS and DFS exhibited satisfactory discrimination and accuracy. Functional enrichment analysis identified that DOK2 was involved in important pathways associated with immune-related activities. Furthermore, CIBERSORT scores reflected negative correlations of DOK2 with activated mast cells and resting CD4+ memory T cells, which indicated its adverse immunomodulatory potential.

**Conclusion:**

We suggest that elevated DOK2 expression could be an unfavorable prognostic indicator of survival in patients with AML. Our findings provide new insights into the role of DOK2 in AML, with promising clinical implications.

## Introduction

Acute myeloid leukemia (AML) is a heterogeneous malignancy of the hematologic system characterized by leukemic cell infiltration of the bone marrow and peripheral blood (1). The incidence of AML and AML-related death have increased rapidly, nearly doubling over the last three decades worldwide (2). The estimated 2-year and 5-year overall survival (OS) rates of AML are 32.0% and 24.0%, respectively, which are markedly lower than those of other leukemia subtypes. Patients diagnosed at age ≥65 years are at extremely high risk, with a median OS (2.67 months) that is the worst of all tumor types (3). Primary driver mutations, co-occurring mutations, and complicated gene-gene interactions account for the heterogeneity of leukemia and affect patients' quality of life; however, there is a lack of consensus regarding standard guidelines for prognosis (4). With the development of sequencing technology and bioinformatics resources, it is of great urgency to discover and evaluate novel markers that provide reliable clinical guidance for diagnostic assessment, survival prediction, and treatment options.

Downstream of tyrosine kinase (DOK) family proteins have been demonstrated to play vital roles in immunoreceptor signaling with adaptor functions. The DOK gene family comprises seven members, DOK1–DOK7, which share similar molecular structures including NH2-terminal pleckstrin homology and phosphotyrosine-binding domains (5). Of these, DOK1 and DOK2 have been more thoroughly studied regarding their biological functions. A previous study revealed that both of DOK1 and DOK2 negatively regulate T cell receptor (TCR) signaling and are involved in the etiology of autoimmune diseases (6). In terms of regulating immune cells, they participate in a negative feedback loop of natural killer cell and CD8+ T cell overactivation (7, 8). Furthermore, DOK1 and DOK2 proteins control myeloid cell cellularity and cell cycle progression in hematopoietic progenitor cells (9). Nonetheless, DOK2 differs from DOK1 in certain ways. DOK2 recruits more Ras GTPase-activating protein (RasGAP) as a downstream protein in CD200 receptor signaling, which is negatively regulated by DOK1 by recruiting CT10 regulated kinase-like (CRKL) (10). They were also found to perform distinct functions in toll-like receptor 2 (TLR2)-induced inflammatory signaling of glial cells (11). Notably, DOK2 is preferentially expressed in myeloid cells and T cells and barely expressed in B cells, while DOK1 is expressed in all three immune cells (5).

In addition to its crucial role in molecular pathways, DOK2 is also associated with the prognosis of several cancers. Previous studies have identified DOK2 as a prognostic predictor in solid tumors such as lung, breast, colorectal, and gastric tumors (12–15). Depletion of DOK2 could inhibit apoptosis induced by chemotherapy and lead to carboplatin resistance in ovarian cancer (16). Additionally, *in vivo* experiments revealed that DOK1 and DOK2 double deficiency in mice triggered myeloproliferative disease similar to human chronic myeloid leukemia (CML) (17, 18). Although DOK2 has been recognized as an indispensable factor in myeloid homeostasis and leukemia pathogenesis (18), few studies have focused on the correlation of DOK2 with prognostic implications in AML patients. The importance of immune function mediated by DOK2 also remains largely unclear.

Therefore, it is essential to fully understand the prognostic and biological roles of DOK2 in AML. We aimed to comprehensively investigate its expression levels and prognostic value in adults with AML. Since it was reported to be involved in immune signaling pathways, we also performed function analyses to provide more evidence for this topic. Moreover, our study was of practical clinical use in therapeutic intervention and drug approaches based on DOK2 expression, supporting it as a robust biomarker for AML therapy.

## Materials and Methods

### Cohort Study

In this retrospective study, patient data were extracted from the Cancer Genome Atlas (TCGA) dataset (19). A total of 139 adult patients with complete sequencing and clinical information were enrolled into the cohort and then divided into two groups according to the median DOK2 expression value. First, we examined the relationship between DOK2 expression and baseline characteristics, including age, mutation, French-American-British (FAB) classification, and other clinical features. Subsequently, survival analysis was performed to determine the prognostic value of DOK2 for overall survival (OS) and disease-free survival (DFS) in the whole cohort and in subgroups. OS was defined as the interval between diagnosis and death due to any cause. DFS was defined as the interval between diagnosis and disease recurrence or progression. Meanwhile, we examined the effects of DOK2 in patients treated with hematopoietic stem cell transplantation (HSCT) or chemotherapy alone. Furthermore, to better predict survival outcomes, significant variables identified in Cox regression analyses were chosen to construct nomograms for OS and DFS. The receiver operating characteristic (ROC) curves with the area under the curve (AUC) were assessed for validity and discrimination of the nomogram models. Calibration plots with 1,000 bootstrap resamples were also created to measure the error between the actual and predicted survival probabilities.

### Public Databases

Genotype-Tissue Expression (GTEx) and TCGA Pan-cancer RNA-seq datasets were retrieved from UCSC Xena (https://xena.ucsc.edu/) (20). The Gene Expression Omnibus (GEO) database (https://www.ncbi.nlm.nih.gov/geo/) was used for external validation. GSE9476 (26 AML and 18 normal CD34+ cells) and GSE30029 (89 AML and 30 normal CD34+ cells) were analyzed for gene expression comparisons between AML samples and healthy controls (21, 22). GSE71014 (104 patient samples) was downloaded to validate the prognostic value of DOK2 (23). The Cancer Cell Line Encyclopedia (CCLE) database (https://sites.broadinstitute.org/ccle/) characterizes mRNA expression and DNA methylation of DOK2 in pan-cancer cell lines, as well as specific AML cell lines (24). The protein-protein interaction (PPI) biological network was predicted using GeneMANIA (http://www.genemania.org) (25). Gene set enrichment analysis (GSEA), co-expression analysis, and target prediction were performed using the LinkedOmics database (http://www.linkedomics.org), which provides a specialized platform for convenient access to multi-omics data from TCGA (26). Correlations of DOK2 with immune-related genes in pan-cancer were presented using the TISIDB database (http://cis.hku.hk/TISIDB) (27). Finally, the influence of DOK2 expression on pharmacologic patterns was described from the Gene Set Cancer Analysis (GSCA) webtool (http://bioinfo.life.hust.edu.cn/GSCA/) (28).

### Function and Immune Analysis

To explore the potential function of genes interacting with DOK2, gene ontology (GO) analyses, including biological process (BP), molecular function (MF), and cellular component (CC), as well as the Kyoto Encyclopedia of Genes and Genomes (KEGG) enrichment analysis, were performed. Differentially expressed mRNAs between the DOK2^high^ and DOK2^low^ groups were screened with an adjusted *P* < 0.05, fold change >2 or <0.5, and then analyzed for GO function and enriched KEGG pathways. The CIBERSORT algorithm was employed to quantify immune cell scores and identify immune cells with significant differences (29). Thereafter, the prognostic influence of these cell types was estimated using univariate analysis, and correlations of DOK2 expression with prognosis-significant immune cell types were exhibited. Additionally, we outlined the relationship between DOK2 and immune-related genes using Spearman's correlation analysis. As further supplementation, the associations between DOK2 expression and immune cell infiltration were evaluated using the ImmuCellAI based on the single-sample GSEA (ssGSEA) algorithm (30), and the relevance between gene set variation analysis (GSVA) enrichment score and immune infiltration was also presented.

### Statistical Analysis

The normality of continuous variables in each group was checked using the Shapiro–Wilk test. The Wilcoxon rank sum test was adopted when the assumption of normal distribution was not verified; otherwise, Student's *t*-test was performed. Categorical variables were compared using the chi-square test or Fisher's exact test. Survival analysis was conducted using the Kaplan–Meier method and assessed using the log-rank test. Univariate and multivariate Cox proportional hazards regression models were used to evaluate the hazard ratio (HR) with corresponding 95% confidence intervals (95% CI). All analyses in the present study were implemented using R software (Version 4.0.2). The results were considered statistically significant when the two-tailed *P* < 0.05.

## Results

### Identification of DOK2 and Characterization of Its Expression

**Figure 1:**
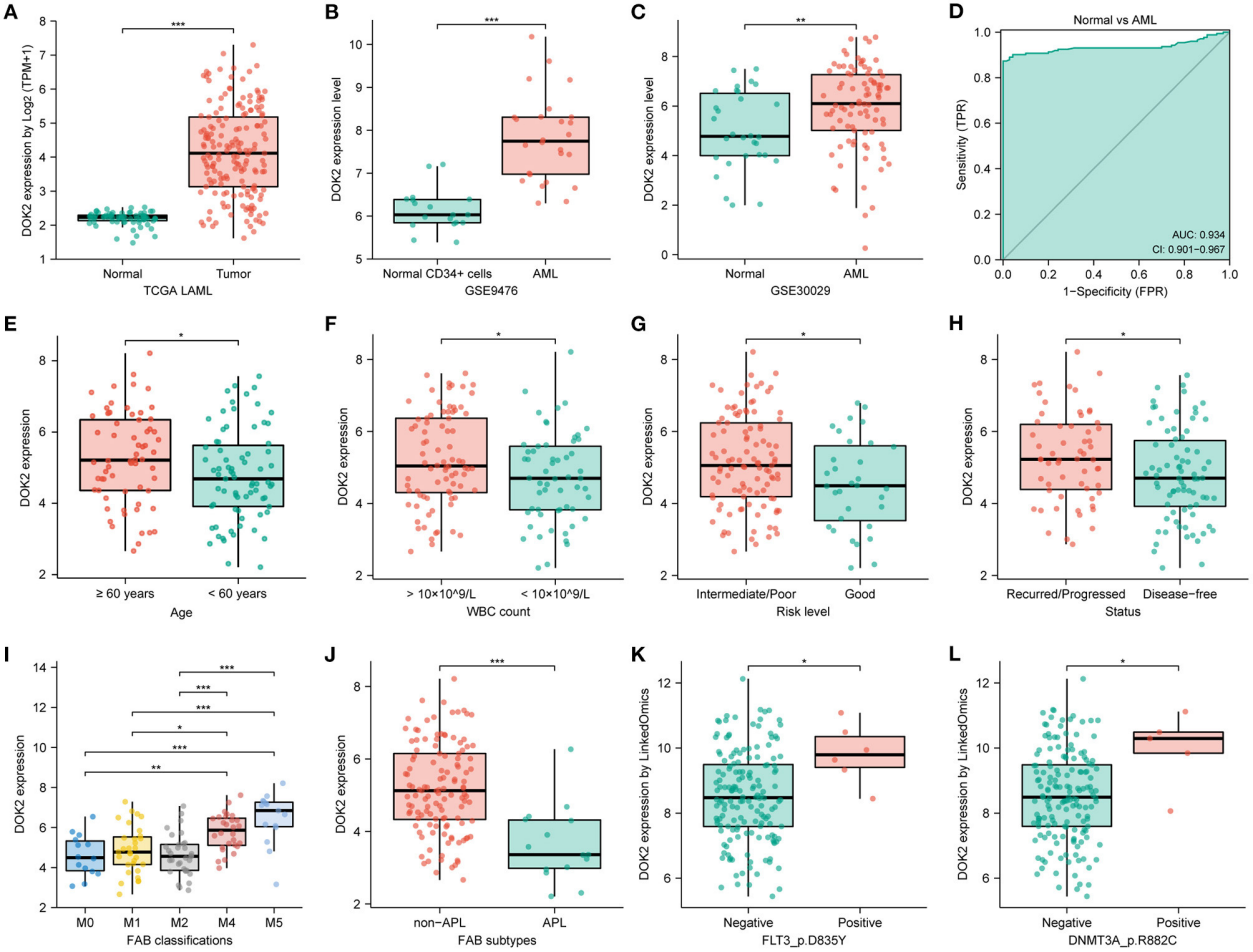
DOK2 mRNA expression levels in AML patients. **(A)** The transcription levels of DOK2 from the TCGA. **(B)** Transcription levels of DOK2 in GSE9476. **(C)** Transcription levels of DOK2 in GSE30029. **(D)** The ROC curve for DOK2 in the TCGA AML vs. normal. **(E–J)** Association of DOK2 expression with age, WBC count, risk level, recurred/progressed status, FAB classification (M0, M1, M2, M4, M5), and APL subtype in the TCGA cohort. **(K,L)** Associations of DOK2 expression with FLT3 p.D835Y mutation and DNMT3A p.R882C mutation by LinkedOmics. ^*^ *P* < 0.05, ^**^ *P* < 0.01, ^***^ *P* < 0.001.

**Figure 2:**
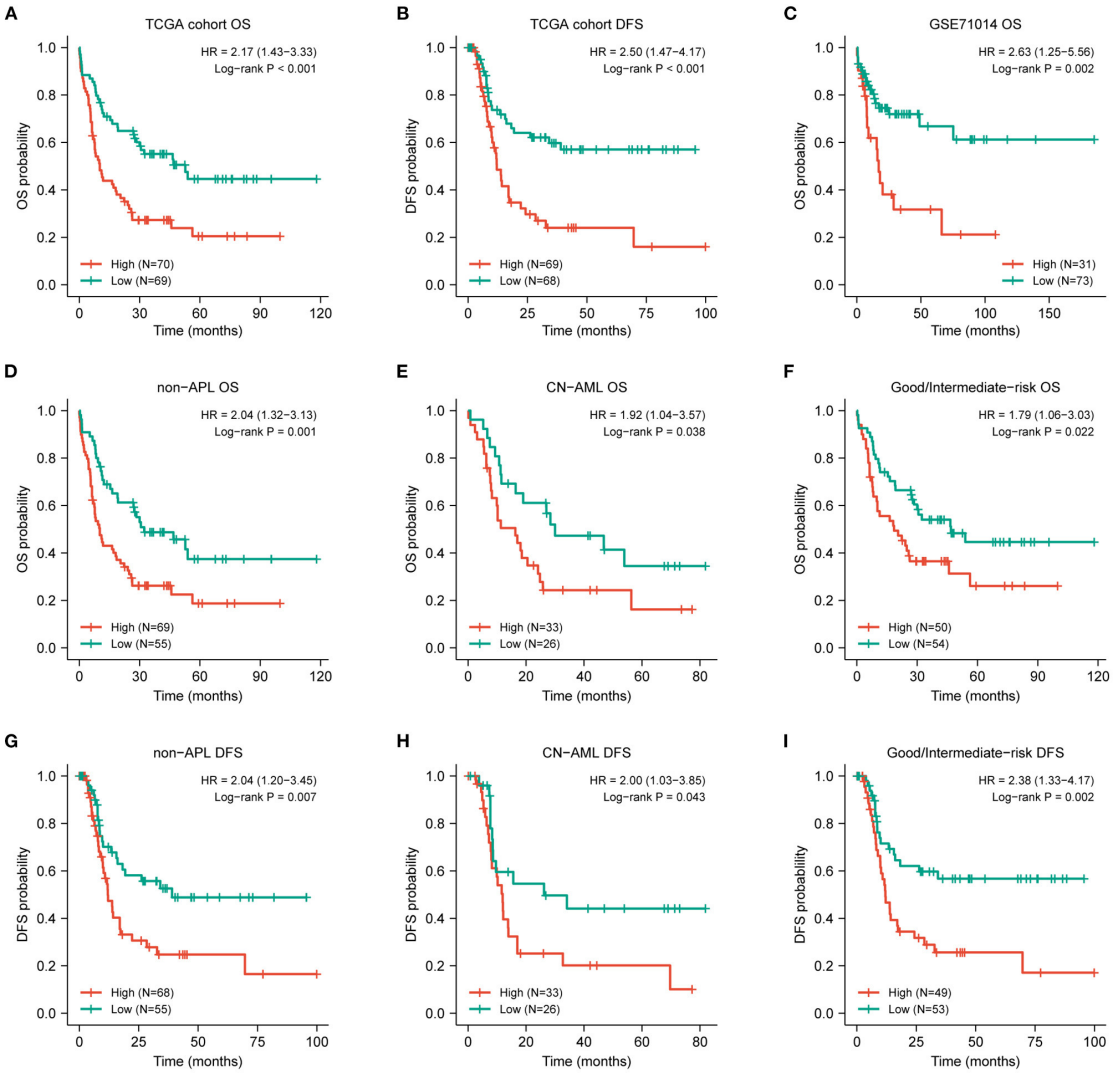
Prognostic value of DOK2 for patients with AML. Kaplan–Meier survival analysis for **(A)** OS and **(B)** DFS in the TCGA cohort. **(C)** Kaplan–Meier survival analysis for AML OS in GSE71014. **(D–F)** Kaplan–Meier curves for OS in non-APL, CN-AML, good/intermediate-risk subsets from the TCGA cohort. **(G–I)** Kaplan–Meier curves for DFS in non-APL, CN-AML, good/intermediate-risk subsets from the TCGA cohort.

We described the expression levels of all DOK gene family members in AML datasets from TCGA (Supplementary Figure S1A). Among them, DOK1, DOK2, DOK3, DOK6, and DOK7 were highly expressed (all *P* < 0.001), whereas DOK4 showed low expression and DOK5 was almost not expressed. DOK1, DOK3, DOK4, DOK6, and DOK7 showed satisfying diagnostic values (Supplementary Figure S2A), yet without prognostic significance in AML (Supplementary Figures S2B–F). Only DOK2 was significant in both differential expression and clinical prognosis (Figures 1A, 2A). Thereby, DOK2 was chosen as the main research subject in the present study.

Moreover, DOK2 positively correlated with DOK1 and DOK3, yet negatively correlated with DOK4 (all *P* < 0.001, Supplementary Figures S1C–E). In AML cell lines, DOK2 expression was highest in OCIAML3 and lowest in HL60 (Supplementary Figure S1B). The TCGA pan-cancer analysis depicted DOK2 mRNA expression in numerous tumors vs. normal tissues (Supplementary Figure S3A). Downregulated DOK2 expression was found in 8 carcinomas (all *P* < 0.001). In contrast, DOK2 expression was upregulated in 10 carcinomas, including AML (all *P* < 0.001). RNA-seq data from the CCLE database revealed that the expression level of DOK2 was highest in AML cell lines, followed by other leukemia types, lymphoma, and multiple myeloma (Supplementary Figure S3B). In contrast to mRNA expression, its DNA methylation levels were significantly lower in AML and other hematologic malignancies among human cancer cell lines (Supplementary Figure S3C). DOK2 expression was significantly higher in AML than in healthy samples (*P* < 0.001; Figure 1A). GSE9476 and GSE30029 datasets confirmed its overexpression in AML cells compared to that in healthy CD34+ cells (*P* < 0.001, Figure 1B; *P* < 0.01, Figure 1C). The ROC curve was analyzed in the TCGA cohort to evaluate its diagnostic value in AML (Figure 1D). The diagnostic efficacy of DOK2 was excellent, with an AUC value of 0.934 (95% CI: 0.901–0.967).

### Associations Between DOK2 and Clinicopathological Characteristics

Clinicopathological information of the 139 patients in the study cohort is shown in Table 1. Patients in the DOK2^high^ group were more likely to be older (*P* = 0.001) and had a higher WBC count (*P* = 0.005). They also tended to be the M4 or M5 subtype, with a higher probability of poor cytogenetic risk level and FLT3, DNMT3A, or RAS mutations. In subgroup analyses, DOK2 expression levels were higher in patients aged ≥60 years (*P* < 0.05) and with white blood cell (WBC) count >10 × 10^9^/L (*P* < 0.05), intermediate/poor risk level (*P* < 0.05), recurred/progressed status (*P* < 0.05), and non-M3 subtypes, especially M4 and M5 (*P* < 0.001) (Figures 1E–J). In addition, the LinkedOmics database implied that DOK2 was highly expressed in the FLT3_p.D835Y mutation and DNMT3A_p.R882C mutation groups at the gene site level (both *P* < 0.05, Figures 1K,L).

**Table 1:** Clinicopathological features of AML patients in the TCGA cohort.

**Characteristics**	**Overall (*N* = 139)**	**DOK2^**low**^ (*N* = 69)**	**DOK2^**high**^ (*N* = 70)**	***P*-value**
Sex (%)				0.556
Male	75 (54.0%)	35 (50.7%)	40 (57.1%)	
Female	64 (46.0%)	34 (49.3%)	30 (42.9%)	
Age (median [IQR])	57.0 [42.5, 67.0]	53.0 [35.0, 63.0]	61.5 [49.2, 71.0]	0.001
WBC count (×10^∧^9/L) (median [IQR])	14.9 [3.8, 46.0]	11.5 [2.5, 34.2]	24.0 [6.5, 58.7]	0.005
BM blasts (%) (median [IQR])	72.0 [52.0, 85.0]	72.0 [56.0, 86.0]	72.0 [48.0, 82.8]	0.219
PB blasts (%) (median [IQR])	37.0 [8.0, 63.0]	41.0 [10.5, 71.5]	22.0 [8.0, 56.0]	0.157
FAB classifications (%)				<0.001
M0	14 (10.1%)	8 (11.6%)	6 (8.6%)	
M1	31 (22.3%)	18 (26.1%)	13 (18.6%)	
M2	33 (23.7%)	21 (30.4%)	12 (17.1%)	
M3	15 (10.8%)	14 (20.3%)	1 (1.4%)	
M4	28 (20.1%)	5 (7.2%)	23 (32.9%)	
M5	15 (10.8%)	2 (2.9%)	13 (18.6%)	
M6	2 (1.4%)	1 (1.4%)	1 (1.4%)	
M7	1 (0.7%)	0 (0.0%)	1 (1.4%)	
FLT3 mutation (%)				0.200
Wild	101 (72.7%)	54 (78.3%)	47 (67.1%)	
Mutated	38 (27.3%)	15 (21.7%)	23 (32.9%)	
DNMT3A mutation (%)				0.453
Wild	106 (76.3%)	55 (79.7%)	51 (72.9%)	
Mutated	33 (23.7%)	14 (20.3%)	19 (27.1%)	
RUNX1 mutation (%)				0.978
Wild	126 (90.6%)	62 (89.9%)	64 (91.4%)	
Mutated	13 (9.4%)	7 (10.1%)	6 (8.6%)	
NPM1 mutation (%)				0.097
Wild	101 (72.7%)	55 (79.7%)	46 (65.7%)	
Mutated	38 (27.3%)	14 (20.3%)	24 (34.3%)	
IDH1 mutation (%)				0.076
Wild	126 (90.6%)	59 (85.5%)	67 (95.7%)	
Mutated	13 (9.4%)	10 (14.5%)	3 (4.3%)	
RAS mutation (%)				0.167
Wild	125 (89.9%)	65 (94.2%)	60 (85.7%)	
Mutated	14 (10.1%)	4 (5.8%)	10 (14.3%)	
Cytogenetics (%)				0.337
Normal	59 (43.1%)	26 (38.2%)	33 (47.8%)	
Abnormal	78 (56.9%)	42 (61.8%)	36 (52.2%)	
Risk level (%)				0.112
Good	32 (23.4%)	21 (30.9%)	11 (15.9%)	
Intermediate	72 (52.6%)	33 (48.5%)	39 (56.5%)	
Poor	33 (24.1%)	14 (20.6%)	19 (27.5%)	

*IQR, interquartile ranges; WBC, white blood cell; BM, bone marrow; PB, peripheral blood; FAB classifications, French-American-British classifications*.

### Survival Curves for OS and DFS

**Figure 3:**
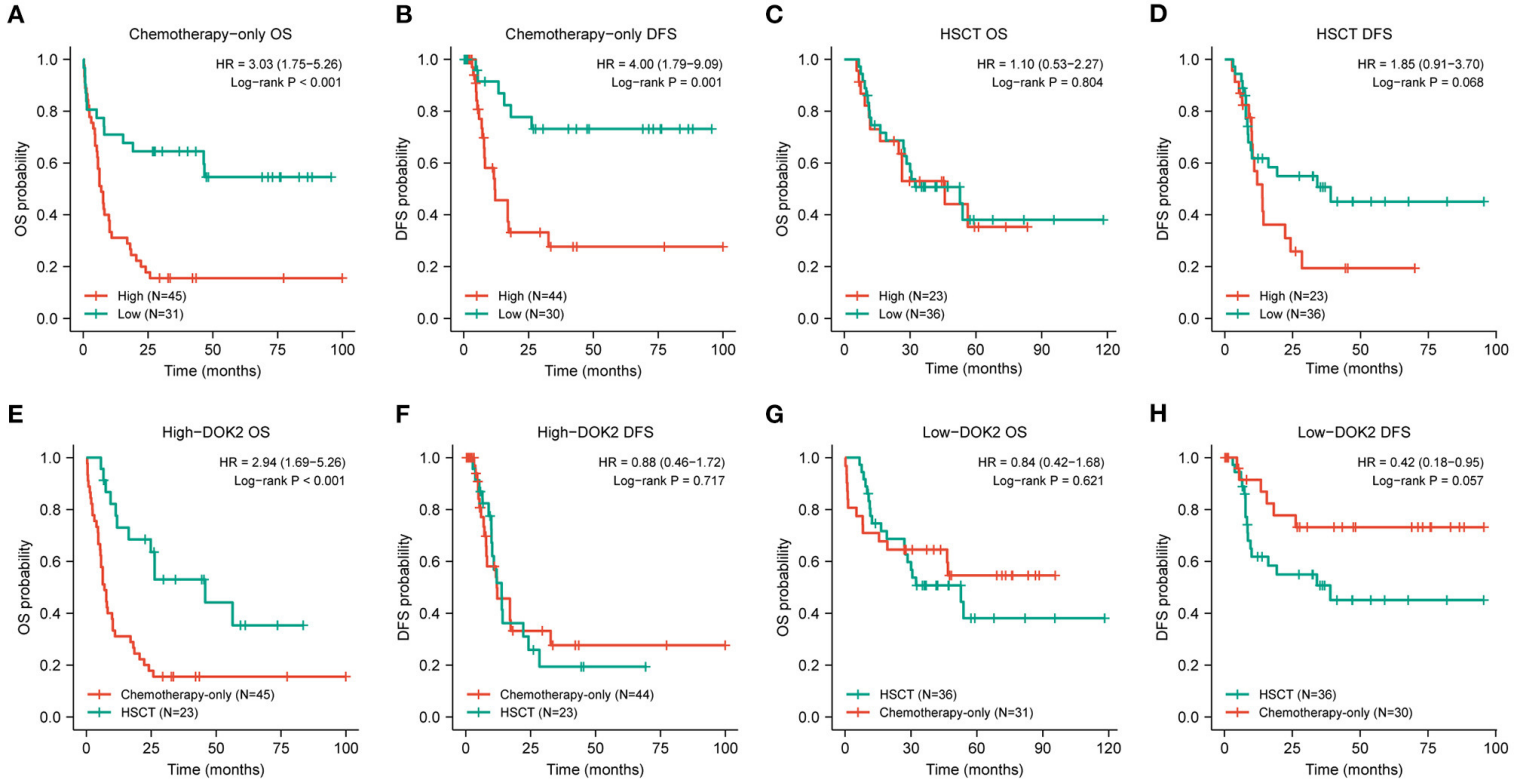
Prognostic value of DOK2 in treatment modalities. **(A,B)** Kaplan–Meier survival analysis for OS and DFS in the chemotherapy-only group. **(C,D)** Kaplan–Meier survival analysis for OS and DFS in the HSCT group. **(E,F)** Comparison of treatment methods for OS and DFS in the DOK2^high^ group. **(G,H)** Comparison of treatment methods for OS and DFS in the DOK2^low^ group.

The expression level of DOK2 was significantly associated with survival outcomes in patients with AML. In the TCGA cohort, high DOK2 expression was related to both inferior OS (*P* < 0.001, Figure 2A) and DFS (*P* < 0.001, Figure 2B). In the GSE71014-GPL10558 dataset, patients with high DOK2 expression had worse OS (cut-off value after log_2_ normalized: 7.235, *P* = 0.002, Figure 2C). Of note, acute promyelocytic leukemia (APL) was associated with improved prognosis and thus excluded from the subgroup analysis. Likewise, unfavorable OS outcomes of the DOK2^high^ group were observed in the non-APL, cytogenetic normal AML (CN-AML), and good/intermediate-risk groups (*P* = 0.001, *P* = 0.038, and *P* = 0.022, respectively; Figures 2D–F), and adverse DFS outcomes of the DOK2^high^ group were also confirmed in the non-APL, CN-AML, and good/intermediate-risk groups (*P* = 0.007, *P* = 0.043, and *P* = 0.002, respectively; Figures 2G–I). For patients who received chemotherapy alone, high DOK2 expression was associated with significantly shorter OS (*P* < 0.001, Figure 3A) and DFS (*P* = 0.001, Figure 3B). However, the influence of DOK2 on survival was no longer significant in patients who received HSCT. In HSCT subgroups, there was no significant effect of DOK2 expression on OS (*P* = 0.804, Figure 3C), and it had less effect on DFS (*P* = 0.068, Figure 3D). We also examined which treatment methods were more favorable for patients based on DOK2 expression levels. Patients with high DOK2 expression benefited more from HSCT than chemotherapy alone with respect to OS (*P* < 0.001, Figure 3E) but not DFS (*P* = 0.717, Figure 3F). In the DOK2^low^ group, there were no significant differences in OS (*P* = 0.621, Figure 3G) or DFS (*P* = 0.057, Figure 3H) between the HSCT and chemotherapy-only groups; however, it was suggested that DOK2^low^ patients could achieve longer DFS through treatment with chemotherapy alone than with HSCT.

### Cox Regression Analyses and Prognostic Nomogram

**Figure 4:**
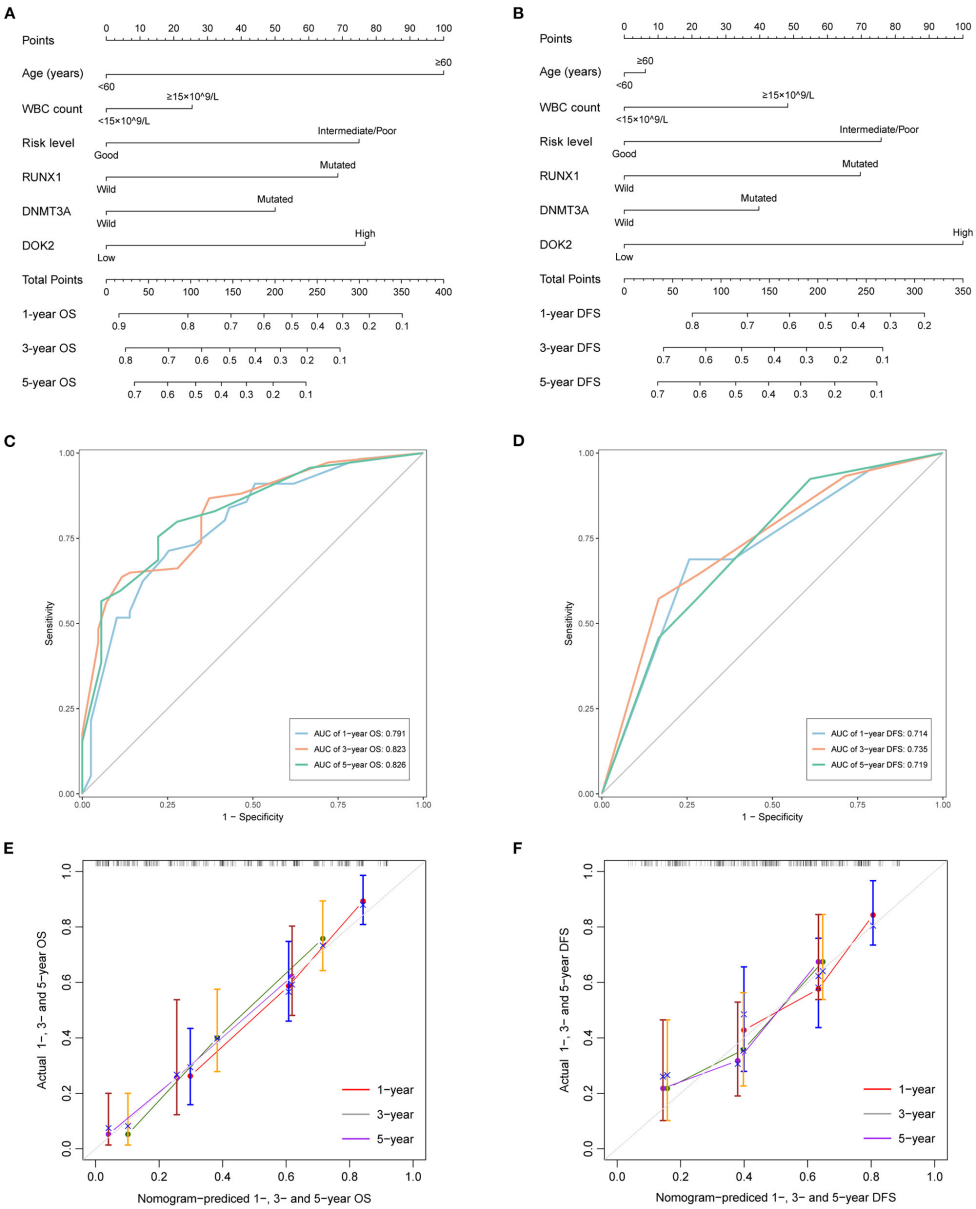
Predictive nomograms for TCGA AML patients. **(A)** Nomogram for predicting 1-, 3-, and 5-year OS. **(B)** Nomogram for predicting 1-, 3-, and 5-year DFS. **(C)** The ROC curve and AUC value of nomogram for OS. **(D)** The ROC curve and AUC value of nomogram for DFS. **(E)** Calibration plots of the nomogram for 1-, 3-, and 5-year OS. **(F)** Calibration plots of the nomogram for 1-, 3-, and 5-year DFS.

As shown in Table 2, univariate analysis showed that age ≥60 years (*P* < 0.001), DNMT3A mutation (*P* < 0.001), RUNX1 mutation (*P* < 0.001), non-good risk level (*P* < 0.001), and high DOK2 expression (*P* < 0.001) were associated with inferior OS; and WBC count ≥15 ×10^9^/L (*P* = 0.024), non-good risk level (*P* = 0.011), and high DOK2 expression (*P* < 0.001) were associated with inferior DFS. Multivariate analysis confirmed that high DOK2 expression was an independent risk factor for both OS and DFS (both *P* = 0.003). Age ≥60 years was also an independent risk factor for OS (*P* < 0.001). Subsequently, a predictive nomogram was established to estimate the 1-, 3-, and 5-year OS and DFS probabilities based on the multivariate Cox analysis (Figures 4A,B). Different categories of clinicopathological factors could be projected onto matching scores, which were summed up to a total score for specific survival rates. The 1-, 3-, and 5-year AUC of the nomogram were 0.791, 0.823, and 0.826, respectively, for OS (Figure 4C), and 0.714, 0.735, and 0.719, respectively, for DFS (Figure 4D), indicating adequate specificity and sensitivity. The calibration plots of 1-, 3-, and 5-year OS and DFS describe the close proximity of the predicted lines to actual reference lines (Figures 4E,F), which guarantees the reliability and accuracy of our nomogram.

**Table 2:** Univariate and multivariate Cox regression analysis for OS and DFS.

**Characteristics**	**Overall survival (OS)**			**Disease-free survival (DFS)**		
	**HR**	**95% CI**	***P*-value**	**HR**	**95% CI**	***P-*value**
**Univariate analysis**						
Age (≥60 years vs. <60 years)	3.253	2.101–5.039	<0.001	1.363	0.807–2.304	0.247
Sex (female vs. male)	0.935	0.611–1.429	0.755	1.046	0.627–1.745	0.863
WBC count (≥15 vs. <15 × 10^∧^9/L)	1.410	0.922–2.157	0.113	1.813	1.081–3.040	0.024
BM blasts (≥70% vs. <70%)	1.149	0.748–1.764	0.526	1.012	0.606–1.690	0.964
PB blasts (≥30% vs. <30%)	1.089	0.709–1.673	0.697	1.600	0.939–2.725	0.084
FLT3 (mutated vs. wild)	1.516	0.954–2.408	0.078	1.451	0.824–2.556	0.198
DNMT3A (mutated vs. wild)	1.905	1.183–3.068	<0.001	1.725	0.964–3.087	0.066
RUNX1 (mutated vs. wild)	2.283	1.225–4.254	<0.001	1.710	0.728–4.018	0.218
NPM1 (mutated vs. wild)	1.257	0.793–1.994	0.330	1.483	0.864–2.547	0.153
IDH1 (mutated vs. wild)	0.746	0.344–1.617	0.458	0.560	0.203–1.546	0.263
RAS (mutated vs. wild)	1.077	0.539–2.152	0.833	1.545	0.701–3.409	0.281
Risk (intermediate/poor vs. good)	3.411	1.756–6.628	<0.001	2.423	1.222–4.805	0.011
DOK2 (high vs. low)	2.241	1.447–3.470	<0.001	2.536	1.493–4.306	<0.001
**Multivariate analysis**						
Age (≥60 years vs. <60 years)	2.504	1.558–4.024	<0.001	1.053	0.593–1.868	0.861
WBC count (≥15 vs. <15 × 10^∧^9/L)	1.263	0.809–1.973	0.305	1.488	0.871–2.544	0.146
DNMT3A (mutated vs. wild)	1.583	0.947–2.646	0.080	1.387	0.742–2.594	0.305
RUNX1 (mutated vs. wild)	1.878	0.968–3.643	0.062	1.775	0.707–4.455	0.222
Risk (intermediate/poor vs. good)	1.989	0.972–4.072	0.060	1.868	0.885–3.941	0.101
DOK2 (high vs. low)	2.023	1.274–3.212	0.003	2.278	1.319–3.935	0.003

*WBC, white blood cell; BM, bone marrow; PB, peripheral blood; HR, hazard ratio; CI, confidence interval*.

### Functional Enrichment and Co-expression Analyses

**Figure 5:**
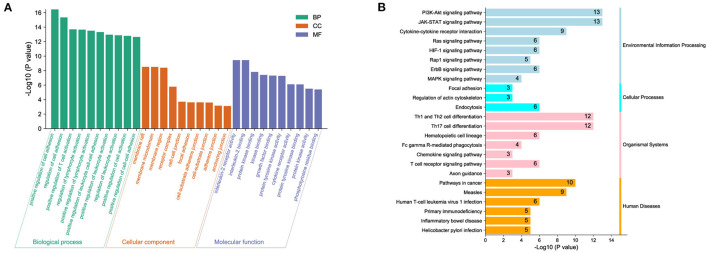
Biological function analysis of DOK2. **(A)** Gene ontology and **(B)** pathway enrichment analysis for DOK2 and its interactive genes. Gene ontology (GO) included biological process (BP), molecular function (MF), and cellular component (CC); KEGG pathways included environmental information processing, cellular processes, organismal systems, and human diseases.

**Figure 6:**
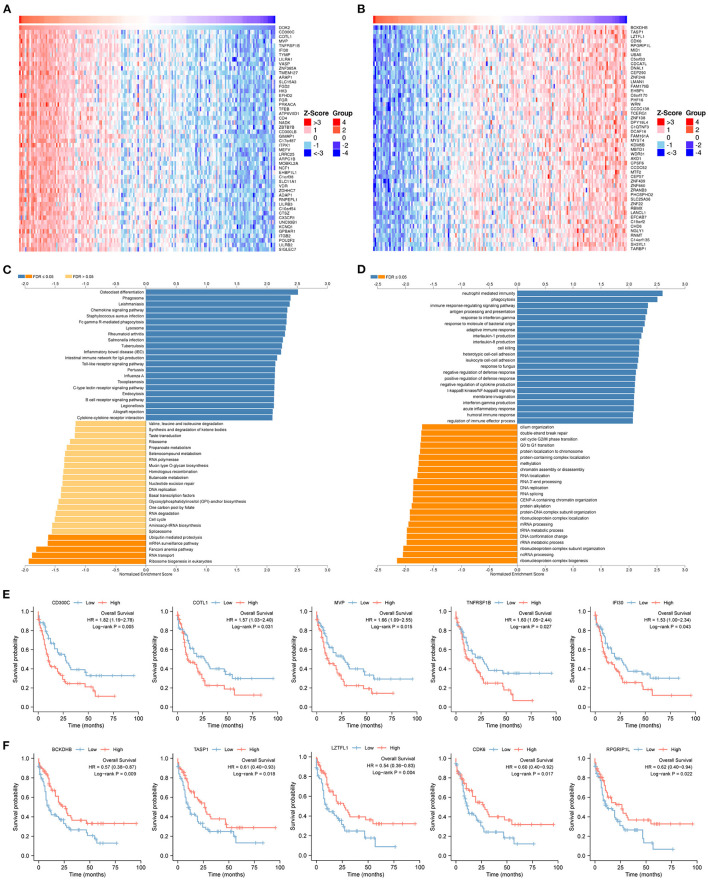
Genes co-expressed with DOK2 according to LinkedOmics. **(A)** Heatmap for top 50 significant genes positively correlated with DOK2 in AML. **(B)** Heatmap for top 50 significant genes negatively correlated with DOK2 in AML. **(C)** GSEA analysis of GO_BP terms analyzed using LinkedOmics. **(D)** GSEA analysis of KEGG terms analyzed using LinkedOmics. **(E)** Prognostic value of top 5 ranked genes positively correlated with DOK2 in the TCGA AML. **(F)** Prognostic value of top 5 ranked genes negatively correlated with DOK2 in the TCGA AML.

To predict biological functions, we first obtained a PPI network for the targets of DOK2 from GeneMANIA (Supplementary Figure S4A). GO enrichment analysis revealed that DOK2 and its interactive genes were involved in the following significant activities (Figure 5A): BP terms included regulation of cell adhesion, T cell activation, and lymphocyte activation; CC terms included membrane raft, microdomain, and region; and MF terms included interleukin-2 receptor activity, interleukin-2 binding, and kinase binding. KEGG enrichment analysis showed the following significant pathways regulated by DOK2 interactive genes (Figure 5B): environmental information processing included the PI3K-Akt signaling pathway, JAK-STAT signaling pathway, and cytokine-cytokine receptor interaction; organismal systems included Th1 and Th2 cell differentiation, Th17 cell differentiation, and hematopoietic cell lineage; and human diseases included pathways in cancer, measles, and human T cell leukemia virus 1 infection. We also identified the differentially expressed genes between the DOK2^high^ and DOK2^low^ groups using a volcano plot (Supplementary Figure S4B and Supplementary Table S1). There were 475 upregulated genes in the DOK2^high^ group, which was much higher than the number of downregulated genes. These upregulated genes participated in tuberculosis, phagosome, and cytokine-cytokine receptor interactions among KEGG pathways (Supplementary Figure S4C); GO terms included neutrophil degranulation and neutrophil activation involved in the immune response (Supplementary Figure S4D). In addition, genes co-expressed with DOK2 were listed in the LinkedOmics database (Supplementary Table S2). The top 50 significantly co-expressed genes that positively and negatively correlated with DOK2 were visualized in the form of heat maps (Figures 6A,B). Notably, high expression of the top five co-expressed genes positively associated with DOK2, including CD300C, COTL1, MVP, TNFRSF1B, and IFI30, was also related to poor prognosis for OS (all *P* < 0.05, Figure 6E). Inversely, high expression of the top five co-expressed genes negatively correlated with DOK2, including BCKDHB, TASP1, LZTFL1, CDK6, and RPGRIP1L, was linked with superior OS (all *P* < 0.05, Figure 6F). GSEA analysis further revealed significant pathway enrichment regulated by DOK2 and its co-expressed genes, such as osteoclast differentiation, neutrophil-mediated immunity, phagocytosis, and immune response-regulating signaling pathways (Figures 6C,D). The most significant kinase target, miRNA target, and transcription factor (TF) target were PRKCD, MIR494, and TF V$SRF_01, respectively (Table 3). Taken together, these results emphasize the important role of DOK2 in immune-related functions.

**Table 3:** Kinase, miRNA and transcription factor targets of DOK2 predicted by LinkedOmics.

**Enriched category**	**Gene set**	**Leading Edge Num**	**NES**	**FDR**
**Kinase targets**	Kinase PRKCD	22	1.9697	0
	Kinase LYN	15	1.9256	0.0030828
	Kinase PRKCA	59	1.8767	0.0030828
	Kinase SYK	12	1.8704	0.0030828
	Kinase ROCK1	20	1.8362	0.0049325
**miRNA targets**	ATGTTTC, MIR-494	39	−1.6557	0.081397
	CAGTCAC, MIR-134	15	−1.6489	0.059110
	CTTGTAT, MIR-381	59	−1.6079	0.082850
	ACCATTT, MIR-522	48	−1.5920	0.079071
	CTGTTAC, MIR-194	25	−1.5631	0.099323
**Transcription factor targets**	V$SRF_01	22	2.1175	0
	V$PU1_Q6	63	1.9789	0
	V$ELF1_Q6	70	1.9569	0
	V$PEA3_Q6	73	1.9280	0.00064293
	RGAGGAARY_V$PU1_Q6	106	1.8957	0.00051434

*NES, normalized enrichment score; FDR, false discovery rate*.

### CIBERSORT and ssGSEA Analyses

**Figure 7:**
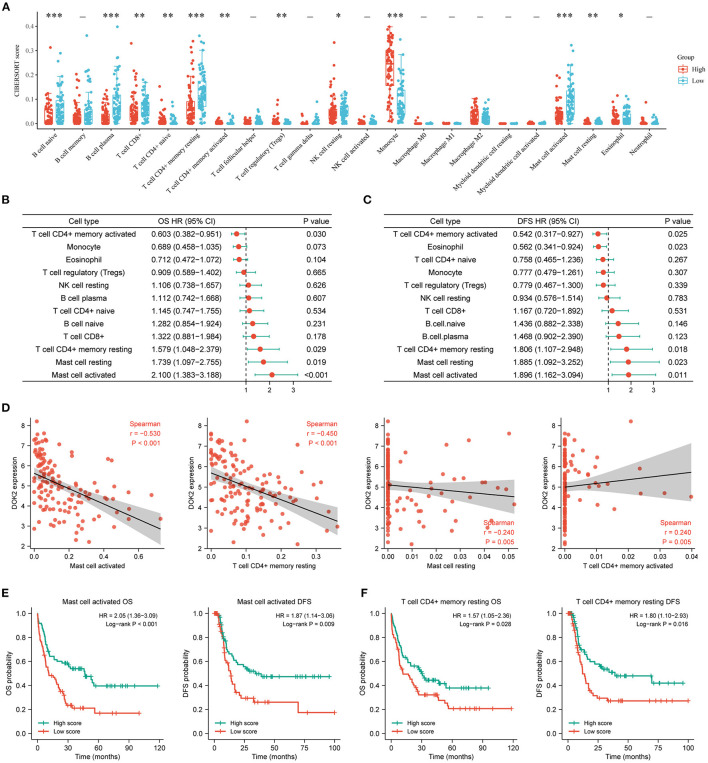
Associations of DOK2 with CIBERSORT immune cell types in TCGA datasets. **(A)** The CIBERSORT score distribution between DOK2^high^ and DOK2^low^ groups. **(B)** Forest plots of significant immune cell types for OS using univariate Cox analysis (low vs. high with high as reference). **(C)** Forest plots of significant immune cell types for DFS using univariate Cox analysis (low vs. high with high as reference). **(D)** Spearman correlation analysis of DOK2 expression with mast cell activated, T cell CD4+ memory resting, mast cell resting, and T cell CD4+ memory activated. **(E)** Kaplan–Meier survival curves for OS and DFS stratified by immune scores of activated mast cells. **(F)** Kaplan–Meier survival curves for OS and DFS stratified by immune scores of resting CD4+ memory T cells. ^*^ *P* < 0.05, ^**^ *P* < 0.01, ^***^ *P* < 0.001.

**Figure 8:**
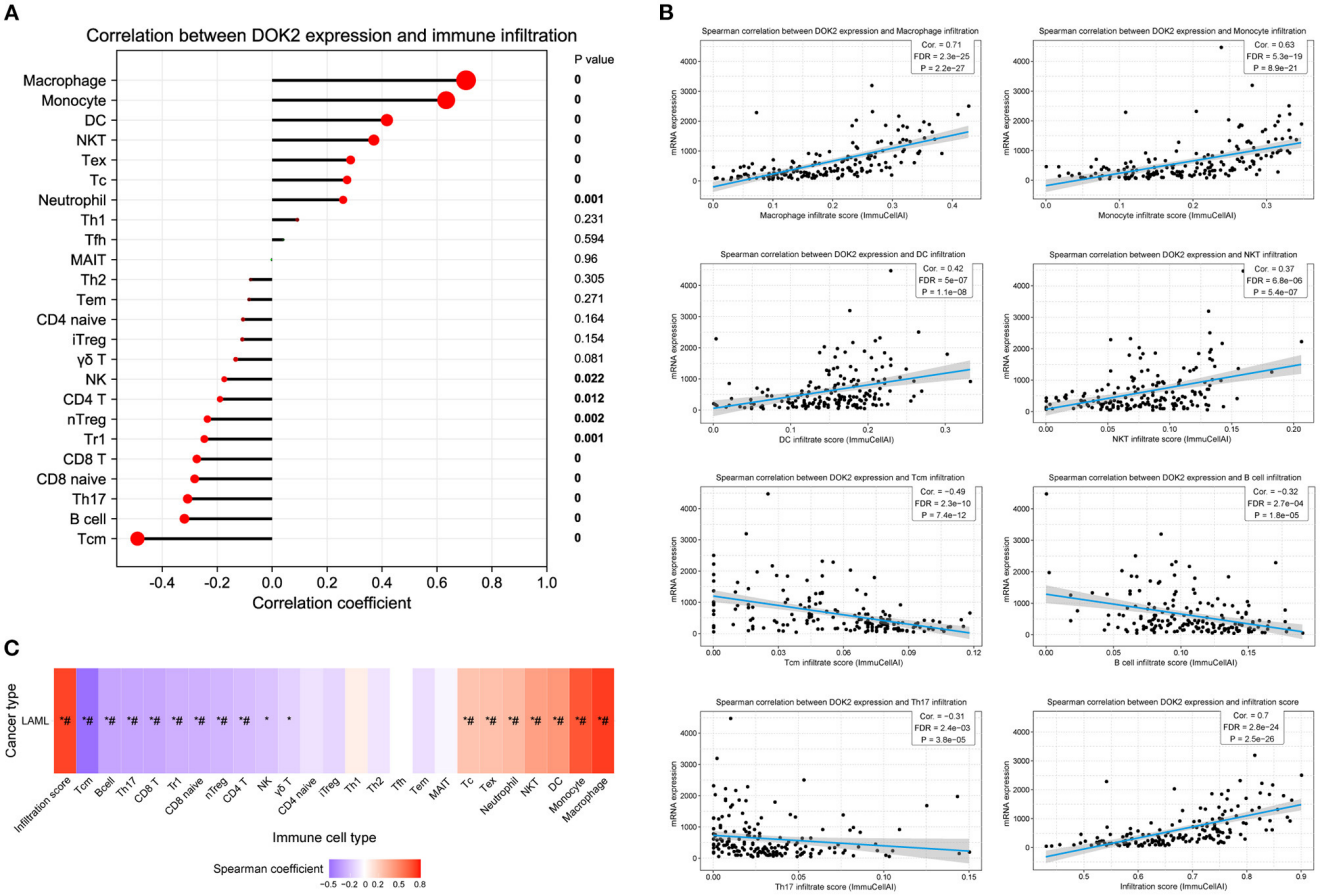
Association between DOK2 expression and immune cell abundance according to GSCA. **(A)** The correlation between DOK2 and immune cell infiltration using ssGSEA. **(B)** Spearman correlations of DOK2 expression with significant immune infiltration types. **(C)** The association between immune cell infiltrates and GSVA score in AML. ^*^ *P* < 0.05; # FDR <0.05.

The CIBERSORT method was applied to score 22 immune cells and evaluate the relationship between DOK2 expression and immune scores (Figure 7A). There were significant differences between the DOK2 high and low groups in the 12 types of immune cells. Samples in each cell type were divided into two groups according to the median value of their immune scores. The prognostic value of these significant cell types was calculated in a univariate analysis based on high and low scores (Figures 7B,C). Low scores of activated mast cells, resting mast cells, and resting CD4+ memory T cells were associated with both worse OS (*P* < 0.001, *P* = 0.019, and *P* = 0.029, respectively) and DFS (*P* = 0.001, *P* = 0.023, and *P* = 0.018, respectively), while a low score of activated CD4+ memory T cells was associated with both better OS (*P* = 0.030) and DFS (*P* = 0.025). As shown in Figure 7D, Spearman correlation analysis revealed that DOK2 expression was negatively associated with immune scores of activated mast cells (r = −0.53, *P* < 0.001), resting CD4+ memory T cells (r = −0.45, *P* < 0.001), and resting mast cells (r = 0.24, *P* = 0.005), but positively associated with immune scores of activated CD4+ memory T cells (r = 0.24, *P* = 0.005). Kaplan–Meier curves showed that patients with low activated mast cell scores had both worse OS and DFS (*P* < 0.001 and *P* = 0.009, respectively, Figure 7E); patients with low resting CD4+ memory T cell scores also had poorer OS and DFS (*P* = 0.028 and *P* = 0.016, respectively, Figure 7F). Moreover, we adopted the ssGSEA algorithm from the GSCA database to provide insights into different types of immune infiltration. As shown in Figure 8A, DOK2 expression significantly positively correlated with the infiltration levels of macrophages, monocytes, dendritic cells (DCs), natural killer T (NKT) cells, exhausted T cells (Tex), and cytotoxic T (Tc) cells (all *P* < 0.001), but negatively correlated with the infiltration levels of central memory T (Tcm) cells, B cells, T helper 17 (Th17) cells, CD8(+) naive (CD8 naive) cells, and CD8(+) T (CD8 T) cells (all *P* < 0.001). Spearman correlations of most associated infiltrates are presented, including microphages, monocytes, DCs, NKT, Tcm, B cells, Th17, and total infiltration score (Figure 8B). The heatmap summarizes the correlations between immune cell infiltration and GSVA enrichment score, which represents the gene set expression level of AML (Figure 8C).

### Associations Between DOK2 and Immune-Related Genes

To deepen our understanding of the interrelationships between DOK2 expression and immune molecules, we selected various immune-related signatures for the exhibition. Spearman correlation analyses revealed that DOK2 positively associated with the expressions of immune checkpoint-related genes, especially HAVCR2, CTLA4, and PDCD1LG2 (Supplementary Figures S5A,B). The correlations of DOK2 with the expressions of chemokines, chemokine receptors, and immunomodulators comprising immune-inhibitors, immune-stimulators, and major histocompatibility complex (MHC) molecules are shown in heat maps (Supplementary Figures S5C–G). We also similarly presented the correlations of DOK2 with these immune-related signatures in pan-cancer from the TISIDB database (Supplementary Figures S6A–E). DOK2 was positively correlated with the majority of these immune markers irrespective of AML or other cancer types, which confirmed its vital role in a wide diversity of immune modulation.

### Drug Sensitivity and DOK2 Expression

Ultimately, we explored whether the expression level of DOK2 could lead to drug resistance or sensitivity. As shown in Supplementary Table S3, the GSCA webtool provided correlations between DOK2 expression level and drug IC50 data from the Genomics of Drug Sensitivity in Cancer (GDSC) database. In Table 4, we outlined the most significant drugs that target DOK2-related pathways or molecules, including PI3K-Akt, JAK-STAT, and MAPK signaling pathways, as well as FLT3. With the increased expression level of DOK2, enhanced sensitivity of cells to these drugs was induced.

**Table 4:** Associations between DOK2 expression and sensitivity to the drugs targeting DOK2-related pathways or molecules.

**Drug name**	**Coefficient**	**FDR**	**Targeted pathway**	**Targeted molecule**
AKT inhibitor VIII	−0.3113744	7.72303E-21	PI3K/MTOR signaling	AKT1, AKT2, AKT3
PIK-93	−0.28775002	6.82289E-18	PI3K/MTOR signaling	PI3K gamma
ZSTK474	−0.23868667	3.44725E-12	PI3K/MTOR signaling	PI3K (class 1)
Idelalisib	−0.23489084	1.11373E-11	PI3K/MTOR signaling	PI3K delta
OSI-027	−0.23208374	1.42746E-11	PI3K/MTOR signaling	MTORC1, MTORC2
AS605240	−0.23549727	7.71227E-11	PI3K/MTOR signaling	PI3K gamma
Omipalisib	−0.18445123	1.69156E-07	PI3K/MTOR signaling	PI3K (class 1)
AZD8055	−0.17713897	2.08109E-06	PI3K/MTOR signaling	MTORC1, MTORC2
YM201636	−0.13839386	0.000096245	PI3K/MTOR signaling	PIKFYVE
PI-103	−0.21903588	2.05765E-10	Other, kinases	PI3K alpha
BIX02189	−0.37194559	8.19171E-30	ERK MAPK signaling	MEK5, ERK5
FR-180204	−0.18272104	2.86258E-06	ERK MAPK signaling	ERK1, ERK2
VX-11e	−0.13552272	0.000789675	ERK MAPK signaling	ERK2
Fedratinib	−0.34937122	2.06258E-26	Other, kinases	JAK2
Ruxolitinib	−0.22701563	5.32964E-10	Other, kinases	JAK1, JAK2
Lestaurtinib	−0.23341325	1.90916E-10	Other, kinases	FLT3, JAK2
Quizartinib	−0.31716711	2.13617E-20	RTK signaling	FLT3
Cabozantinib	−0.27791877	3.0146E-15	RTK signaling	FLT3
Sunitinib	−0.32753516	7.14689E-09	RTK signaling	FLT3
WZ3105	−0.26093834	1.17337E-14	Other	FLT3

*FDR, false discovery rate*.

## Discussion

Despite the importance of DOK2 in immune-related pathways, there remains a paucity of evidence regarding its influence on AML prognosis and immunology. Hence, it is imperative to provide more evidence on the issue based on multi-platform approaches. For the first time, we described the aberrant expression and dismal prognosis in AML patients. Biological analyses revealed the diverse immune functions of DOK2 and its interactive or co-expressed genes. In addition, DOK2 was strongly associated with multiple immune cell infiltrations, which accounted for poor survival. The effects of DOK2 expression on treatment outcomes cannot be ignored, and therapeutic strategies should be designed individually.

In the present study, DOK2 expression exhibited heterogeneity in pan-cancer analyses, which implied its inconsistent roles in different tissues. For instance, the downregulation of DOK2 expression in lung and colorectal cancer suggests its role as a tumor suppressor (12, 14), while high DOK2 expression was found in ovarian cancer and melanoma. Herein, we noticed that DOK2 was conspicuously overexpressed in AML cell lines and other hematologic neoplasms, with significant hypomethylation levels. In addition, higher expression levels of DOK2 were observed in AML patient samples than in their healthy counterparts. We therefore speculated that its elevated expression in AML was not only a preferential expression in myeloid cells, but also a tumor-associated dysregulation. The excellent diagnostic value of DOK2 was proven by its high AUC value, suggesting its potential for clinical applications.

DOK2 was previously reported to exert inhibitory effects on solid tumors and CML-like disease (12–14, 16, 17). However, we discovered that high DOK2 expression was significantly associated with worse OS and DFS in AML patients. The adverse prognostic effect was not weakened in CN-AML and non-poor risk subgroups, which were recognized as appropriate types with fewer chromosomal and molecular abnormalities for biomarker identification. In addition, the prognostic effect was not subject to the APL subtype. These findings indicate the discriminatory value of DOK2 in AML survival outcomes. We hypothesize that DOK2 may play varying roles in different cancers, leading to opposing survival trends. Of note, our results were not consistent with those of another similar study (31), which could be attributed to disparities in race, regions, treatment methods, disease status, and detection methods. This inconsistency requires further research to address. Indeed, this is where the limitations of our study lie. External clinical samples and basic experimental research are needed to provide stronger support.

The relationship between DOK2 expression and clinical features is worthy of attention. High expression of DOK2 was related to older age, which served as a significant risk factor in AML survival (32). Patients with high DOK2 levels were characterized by high WBC counts, probably due to the numerous immune activities mediated by DOK2 in GSEA analysis (for example, neutrophil-mediated immunity). Patients with disease recurrence or progression were associated with high expression levels of DOK2, and the effects of DOK2 on DFS seemed more notable than those on OS, indicating that DOK2 potentially plays a distinct role in relapse processes. In relation to FAB classifications, non-APL patients had higher DOK2 expression than APL patients, which might explain their poorer survival. Moreover, higher DOK2 expression levels were observed in M4 and M5 (acute monocytic leukemia) than in other FAB subtypes. Monocyte infiltration levels were significantly higher in the DOK2^high^ group in CIBERSORT analysis. The results of ssGSEA also delineated the significant positive correlations between DOK2 and macrophages/monocyte infiltration, providing potential evidence of leukemogenesis in the AML monocytic lineage. Furthermore, DOK2 expression appears to be abnormally upregulated in FLT3 p.D835Y and DNMT3A p.R882C somatic mutations. Both FLT3 and DNMT3A mutations generally have the clinical relevance to reduced survival in AML (4, 33).

We performed functional analyses of multiple aspects to determine the underlying biological mechanisms. As expected, DOK2 with its interactive or co-expressed genes, is involved in a broad range of immunological activities. Pathway analysis confirmed its significant involvement in TCR signaling, and this was consistent with the findings of a previous study (6). TCR-based immunotherapy is an emerging technique after chimeric antigen receptor T (CAR-T) cell therapy, and DOK2 as a negative regulator, may contribute to a better understanding of the mechanisms of TCR resistance (34). Additionally, DOK2 was found to regulate the differentiation of Th17 cells, as supported by the negative correlation of DOK2 with Th17 cell infiltrates from ssGSEA. The KEGG results also highlighted the enriched pathways mediated by DOK2, such as PI3K-Akt, JAK-STAT, Ras, and MAPK signaling pathways. These pathways have been extensively studied in human cancers for targeted anticancer drug therapies. For instance, the PI3K-Akt pathway is considered hyperactivated in AML to sustain leukemic progression, and corresponding inhibitors can prevent the development of leukemia cells (35, 36). Increased activity of JAK/STAT signaling has been shown to sustain the growth and progression of leukemia stem cells, especially in high-risk AML. Aberrant JAK/STAT signaling activity could be repressed by inhibiting FLT3 (37). We utilized the GDSC data to enumerate the targeted drugs against relevant molecules in these pathways and evaluated their correlations with DOK2 expression. High expression of DOK2 increased sensitivity to the listed drugs (Table 4), which underscored their pharmacological value in DOK2-related targets with clinical effectiveness.

We then focused on the correlations of DOK2 expression with various immune markers and immune infiltration levels. The positive correlations between DOK2 and immune checkpoint genes, including HAVCR2, PD-L2, and CTLA4 among others, could be potential mechanisms for immune escape and immunotherapy in AML. The vast majority of immune-related genes are strongly correlated with DOK2 in AML and other cancers, suggesting a wide variety of effects on the immunological activities of the tumor microenvironment. The infiltration score has a strong positive relationship with DOK2, implicating the participation of DOK2 in immune cell infiltration of the bone marrow microenvironment. Infiltrating mast cells have been identified as a biomarker of poor survival and an immunotherapy target in AML (38). We confirmed that the infiltration level of activated or resting mast cells, downregulated by DOK2, resulted in the shorter survival of patients. Curiously, we found a phenomenon concerning HSCT therapy where HSCT was effective in patients with high DOK2 expression in improving OS but showed little efficacy regarding DFS. Here, we found that DOK2 inversely correlated with the infiltration of central memory T cells. Exhaustion of bone marrow central memory T cells could restrict the TCR repertoire, impede effector functions, and exacerbate leukemia burden, thus resulting in early relapse after HSCT (39). Additionally, the low infiltration level of resting CD4+ memory T cells correlated with high DOK2 expression, conferring poor patient outcomes. As reviewed above, these findings indicate that DOK2 could be associated with infiltrating immune cells in the leukemia microenvironment and render poor prognosis as mediated by specific immune cell infiltration, which is anticipated to be a new immune-related therapeutic target in AML.

Given the considerable significance of DOK2 in AML patients, we sought to build a pragmatic nomogram for clinicians and patients to accurately predict survival outcomes. Multivariate analysis verified DOK2 as an independent prognostic factor in addition to patient age. Nomograms indicated that the effects of DOK2 were more significant than almost every other factor in the prediction of OS and DFS. Our nomograms are of practical applicability with satisfactory accuracy and discrimination, which will provide helpful guidance in actual clinical decisions. More importantly, DOK2 is highly instructive for treatment options in patients with AML. For patients undergoing chemotherapy without HSCT, high DOK2 levels were associated with worse survival outcomes. Nonetheless, this influence caused by high DOK2 expression could be overcome by HSCT. Patients receiving HSCT showed no significant differences in survival between the DOK2^high^ and DOK2^low^ groups. However, patients characterized by high DOK2 levels would gain greater benefit from HSCT than chemotherapy alone with respect to OS. In contrast, patients with low DOK2 levels were more likely to prolong their DFS by receiving chemotherapy alone. Consequently, appropriate treatment regimens should be selected according to the DOK2 expression level.

In summary, DOK2 was determined to be an independent prognostic factor for significant survival disparities in AML. We also elucidated its underlying immunologic importance, especially its correlation with immune cell infiltration. Based on the DOK2 expression level, prognostic nomograms can better direct precise prediction, and therapies can be well planned for individualized treatment. Our investigation contributes to further understanding of oncological mechanisms and immunotherapies for AML.

## Data Availability Statement

The datasets presented in this study can be found in online repositories. The names of the repository/repositories and accession number(s) can be found in the article/Supplementary Material.

## Author Contributions

BC and XD: conceptualization, supervision, and writing – review and editing. JX: formal analysis, methodology, and writing – original draft. RW: data curation and resources. All authors have read and approved the manuscript.

## Funding

This work was supported by the grants from the Jiangsu Provincial Medical Innovation Team (CXTDA2017046) and the Nanjing Medical Science and Technique Development Foundation (YKK20083).

## Conflict of Interest

The authors declare that the research was conducted in the absence of any commercial or financial relationships that could be construed as a potential conflict of interest.

## Publisher's Note

All claims expressed in this article are solely those of the authors and do not necessarily represent those of their affiliated organizations, or those of the publisher, the editors and the reviewers. Any product that may be evaluated in this article, or claim that may be made by its manufacturer, is not guaranteed or endorsed by the publisher.

## Supplementary Material

Supplementary Figure S1 Expression of DOK2 and other DOK genes in AML. **(A)** The expression boxplots of DOK family members in AML compared with healthy controls. **(B)** DOK2 expression values in AML cell lines obtained from the CCLE. **(C–E)** Spearman correlations of DOK2 with expression levels of DOK1, DOK3, and DOK4 in AML. ^***^*P* < 0.001.

Supplementary Figure S2 Diagnostic efficacy and prognostic value of other DOK genes in AML. **(A)** The ROC curves for other DOK family members in the TCGA AML vs. normal. Kaplan-Meier survival analysis of **(B)** DOK1, **(C)** DOK3, **(D)** DOK4, **(E)** DOK6, and **(F)** DOK7 for OS in the TCGA cohort.

Supplementary Figure S3 DOK2 expression levels in pan-cancer analysis. **(A)** Boxplots for DOK2 expression in pan-cancer compared with healthy controls. **(B)** The mRNA expression levels of DOK2 within diverse cancer cell lines from the CCLE. **(C)** The DNA methylation levels of DOK2 in different cancer cell lines from the CCLE. The interpretation of cancer abbreviations can be obtained from the TCGA data portal (https://portal.gdc.cancer.gov).

Supplementary Figure S4 Interactive genes with DOK2 and analysis of differentially expressed genes. **(A)** Protein–protein interaction network of DOK2 according to GeneMANIA. **(B)** Volcano plot for differential gene expression profiles between DOK2^high^ and DOK2^low^ groups in the TCGA cohort. **(C)** Kyoto Encyclopedia of Genes and Genomes (KEGG) and gene ontology (GO) enrichment analysis for up-regulated genes in the volcano plot.

Supplementary Figure S5 Spearman correlation analysis between DOK2 and immune-related genes in the TCGA cohort. **(A,B)** Spearman correlations between DOK2 and immune checkpoint-related genes (HAVCR2, CTLA4, PDCD1LG2, LAG3, CD274, and PDCD1). **(C)** Spearman correlations between DOK2 and immune stimulators. **(D)** Spearman correlations between DOK2 and chemokines. **(E)** Spearman correlations between DOK2 and MHC molecules. **(F)** Spearman correlations between DOK2 and immune inhibitors. **(G)** Spearman correlations between DOK2 and chemokine receptors.

Supplementary Figure S6 Spearman correlations between DOK2 and immune-related genes in pan-cancer in TISIDB. **(A)** Spearman correlations between DOK2 and immune stimulators. **(B)** Spearman correlations between DOK2 and chemokines. **(C)** Spearman correlations between DOK2 and immune inhibitors. **(D)** Spearman correlations between DOK2 and chemokine receptors. **(E)** Spearman correlations between DOK2 and MHC molecules.

## Abbreviations

AML: Acute myeloid leukemia
OS: Overall survival
DOK: Downstream of tyrosine kinase
TCR: T cell receptor
CML: Chronic myeloid leukemia
TCGA: The Cancer Genome Atlas
FAB: French-American-British classification
DFS: Disease-free survival
HSCT: Hematopoietic stem cell transplantation
ROC: Receiver operating characteristic
AUC: Area under the curve
GEO: Gene Expression Omnibus
CCLE: Cancer Cell Line Encyclopedia
PPI: Protein–protein interaction
GSEA: Gene set enrichment analysis
GSCA: Gene Set Cancer Analysis
GO: Gene ontology
BP: Biological process
MF: Molecular function
CC: Cellular component
KEGG: Kyoto Encyclopedia of Genes and Genomes
ssGSEA: single-sample GSEA
GSVA: Gene set variation analysis
HR: Hazard ratio
CI: Confidence interval
WBC: White blood cell
APL: Acute promyelocytic leukemia
CN-AML: Cytogenetic normal AML
TF: Transcription factor
DC: Dendritic cells
NKT: Natural killer T
Tcm: Central memory T
Th17: T helper 17
GDSC: Genomics of Drug Sensitivity in Cancer.
