# Supplementary figures and images for: DOK2 Has Prognostic and Immunologic Significance in Adults With Acute Myeloid Leukemia: A Novel Immune-Related Therapeutic Target
Source: Front Med (Lausanne). 2022 Mar 7;9:842383. doi: 10.3389/fmed.2022.842383 (PMC8935080; doi:10.3389/fmed.2022.842383)

**A**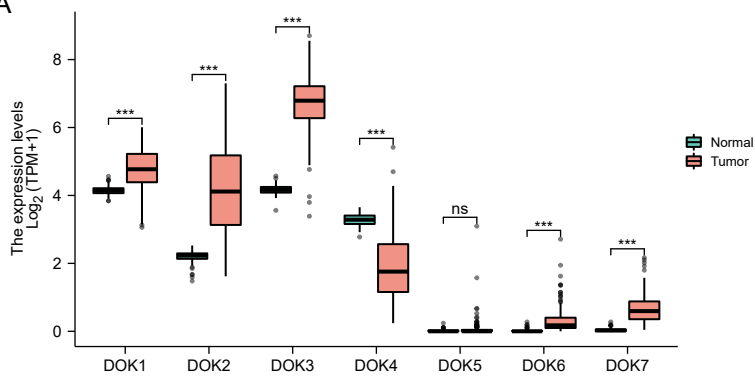**C**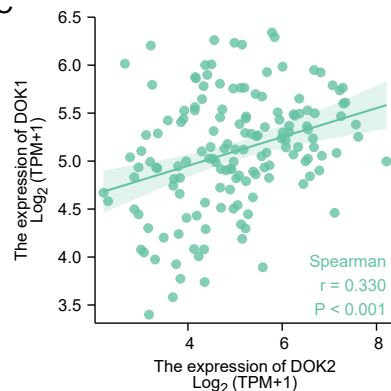**B**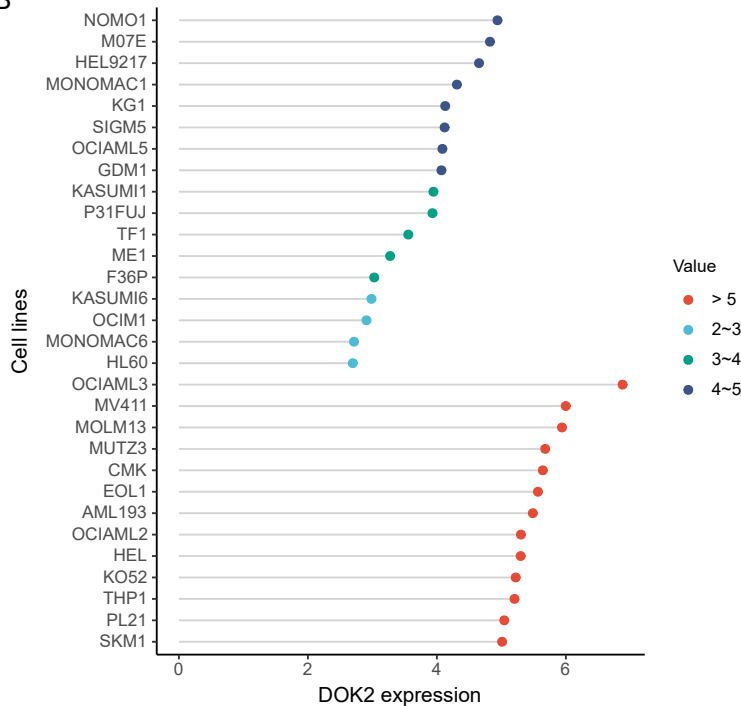**D**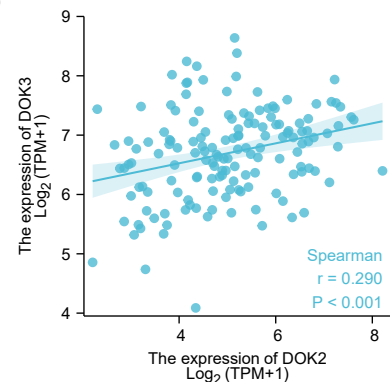**E**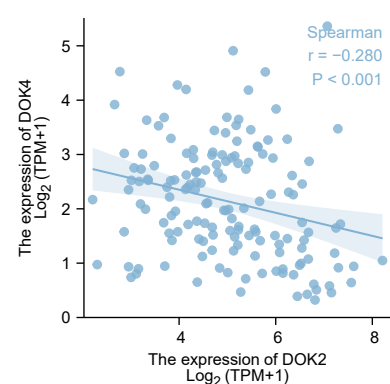

Supplement: Supplementary Figure S1 — Expression of DOK2 and other DOK genes in AML. (A) The expression boxplots of DOK family members in AML compared with healthy controls. (B) DOK2 expression values in AML cell lines obtained from the CCLE. (C–E) Spearman correlations of DOK2 with expression levels of DOK1, DOK3, and DOK4 in AML. ***P < 0.001. [file Data_Sheet_1.pdf]

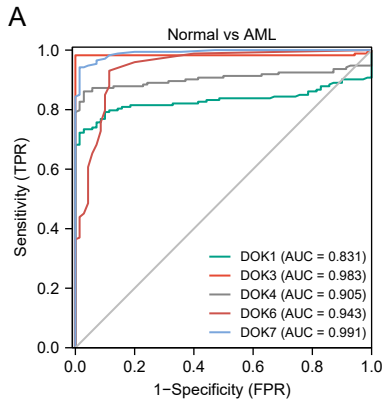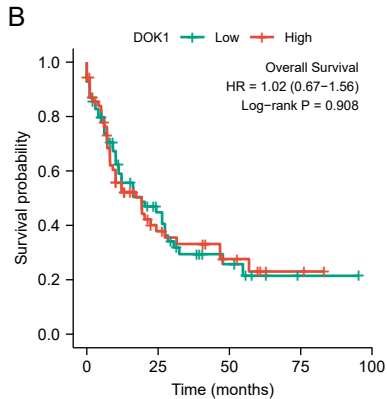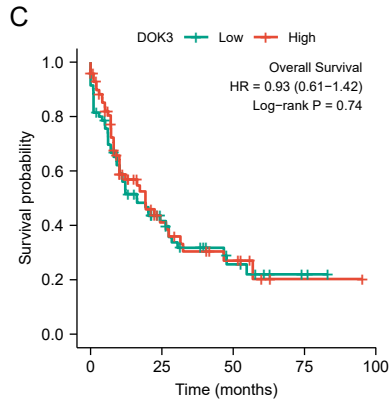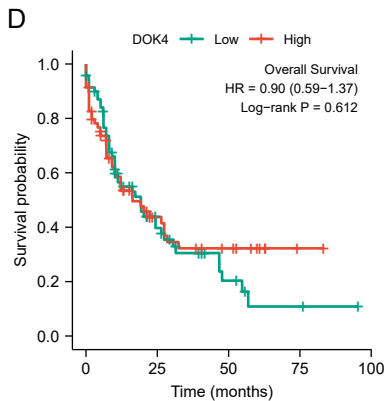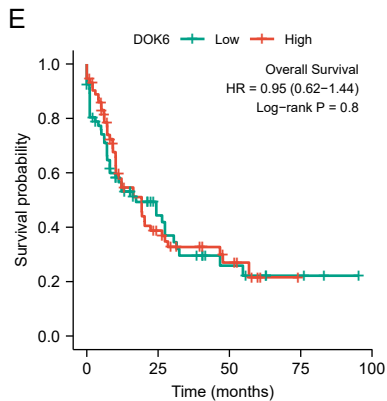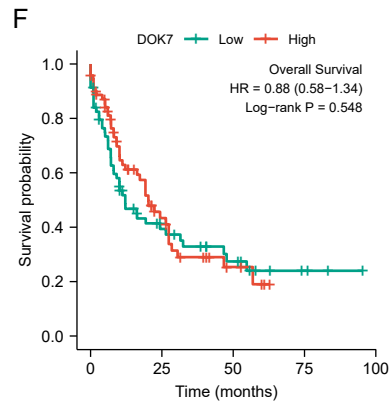

Supplement: Supplementary Figure S2 — Diagnostic efficacy and prognostic value of other DOK genes in AML. (A) The ROC curves for other DOK family members in the TCGA AML vs. normal. Kaplan-Meier survival analysis of (B) DOK1, (C) DOK3, (D) DOK4, (E) DOK6, and (F) DOK7 for OS in the TCGA cohort. [file Data_Sheet_2.pdf]

A

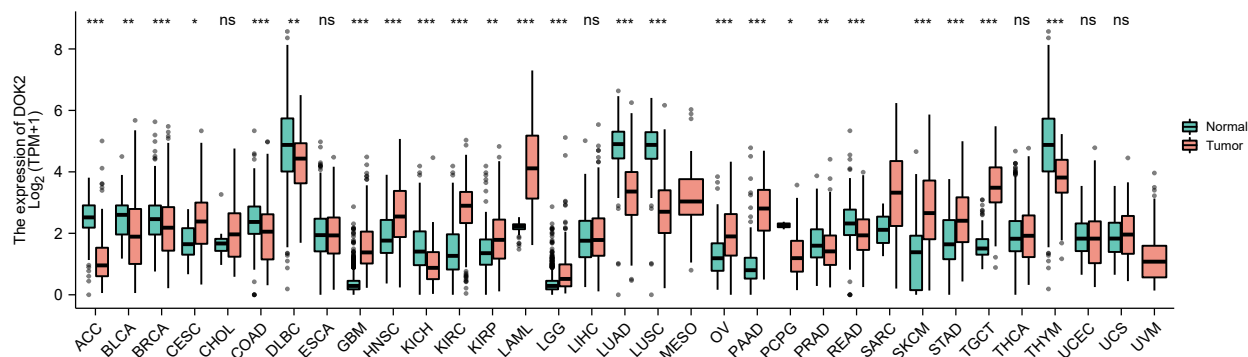

B

mRNA expression (RNAseq): DOK2

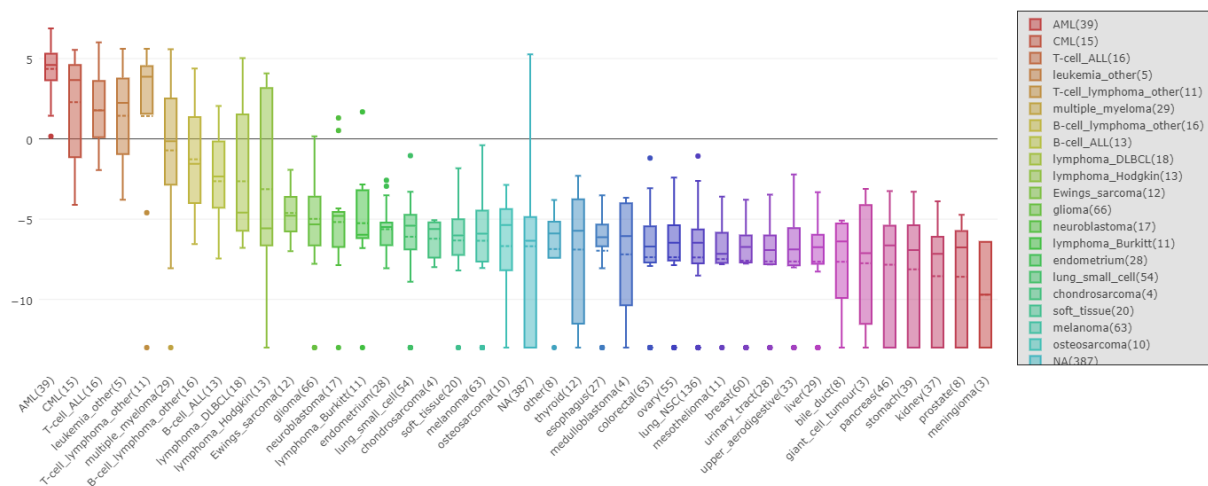

C

DNA methylation (RRBS): DOK2

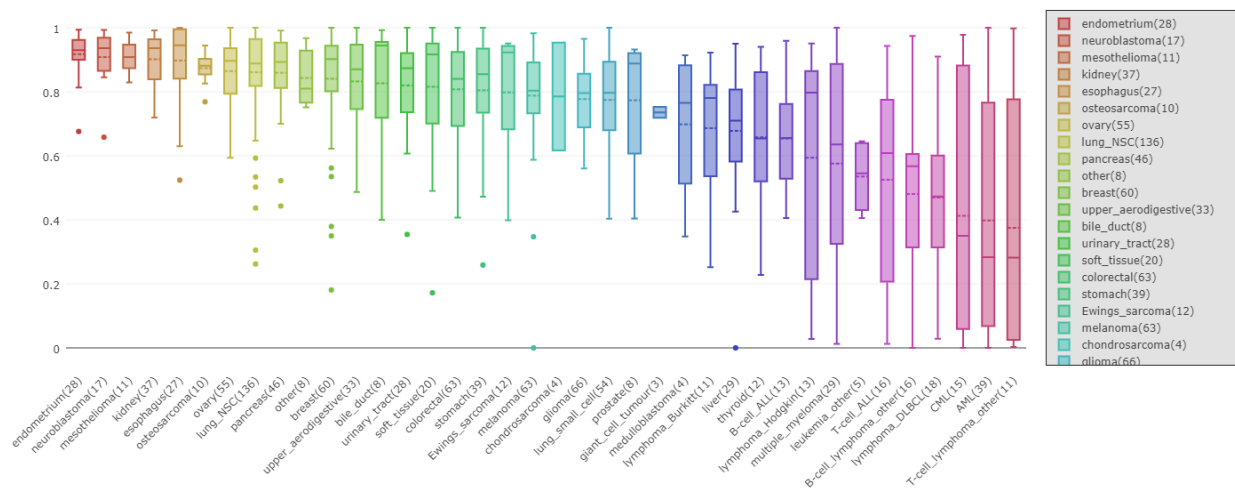

Supplement: Supplementary Figure S3 — DOK2 expression levels in pan-cancer analysis. (A) Boxplots for DOK2 expression in pan-cancer compared with healthy controls. (B) The mRNA expression levels of DOK2 within diverse cancer cell lines from the CCLE. (C) The DNA methylation levels of DOK2 in different cancer cell lines from the CCLE. The interpretation of cancer abbreviations can be obtained from the TCGA data portal (https://portal.gdc.cancer.gov). [file Data_Sheet_3.pdf]

A

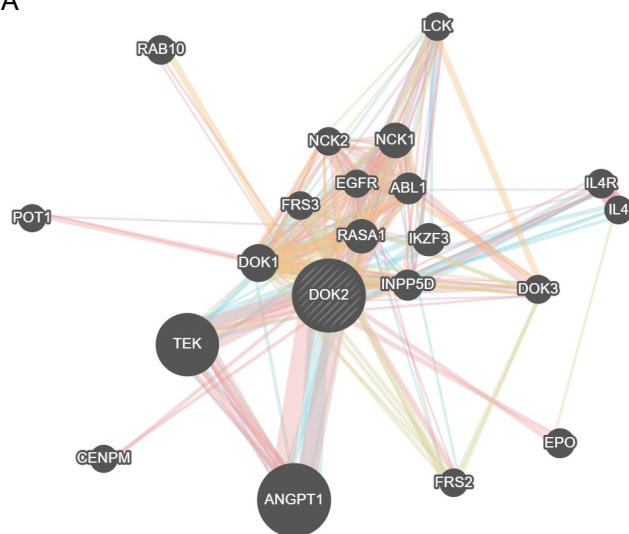

B

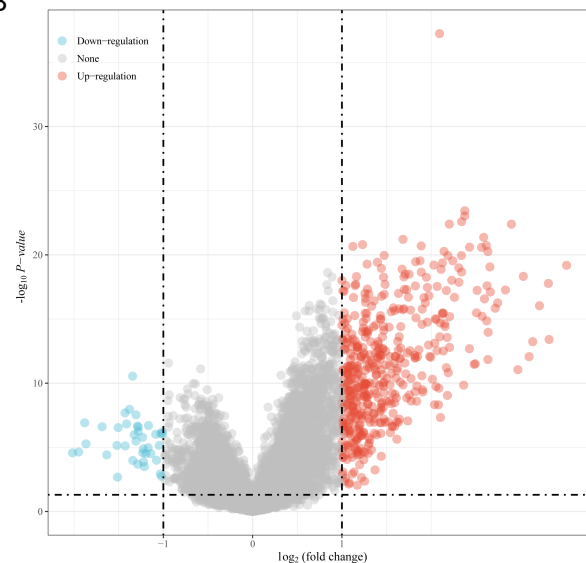

C

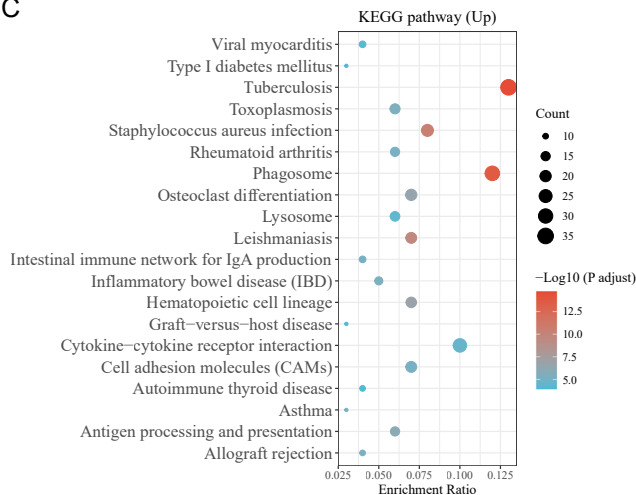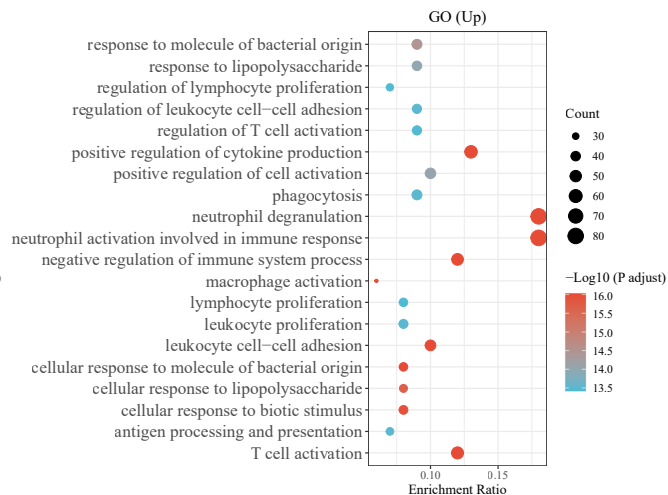

Supplement: Supplementary Figure S4 — Interactive genes with DOK2 and analysis of differentially expressed genes. (A) Protein–protein interaction network of DOK2 according to GeneMANIA. (B) Volcano plot for differential gene expression profiles between DOK2high and DOK2low groups in the TCGA cohort. (C) Kyoto Encyclopedia of Genes and Genomes (KEGG) and gene ontology (GO) enrichment analysis for up-regulated genes in the volcano plot. [file Data_Sheet_4.pdf]

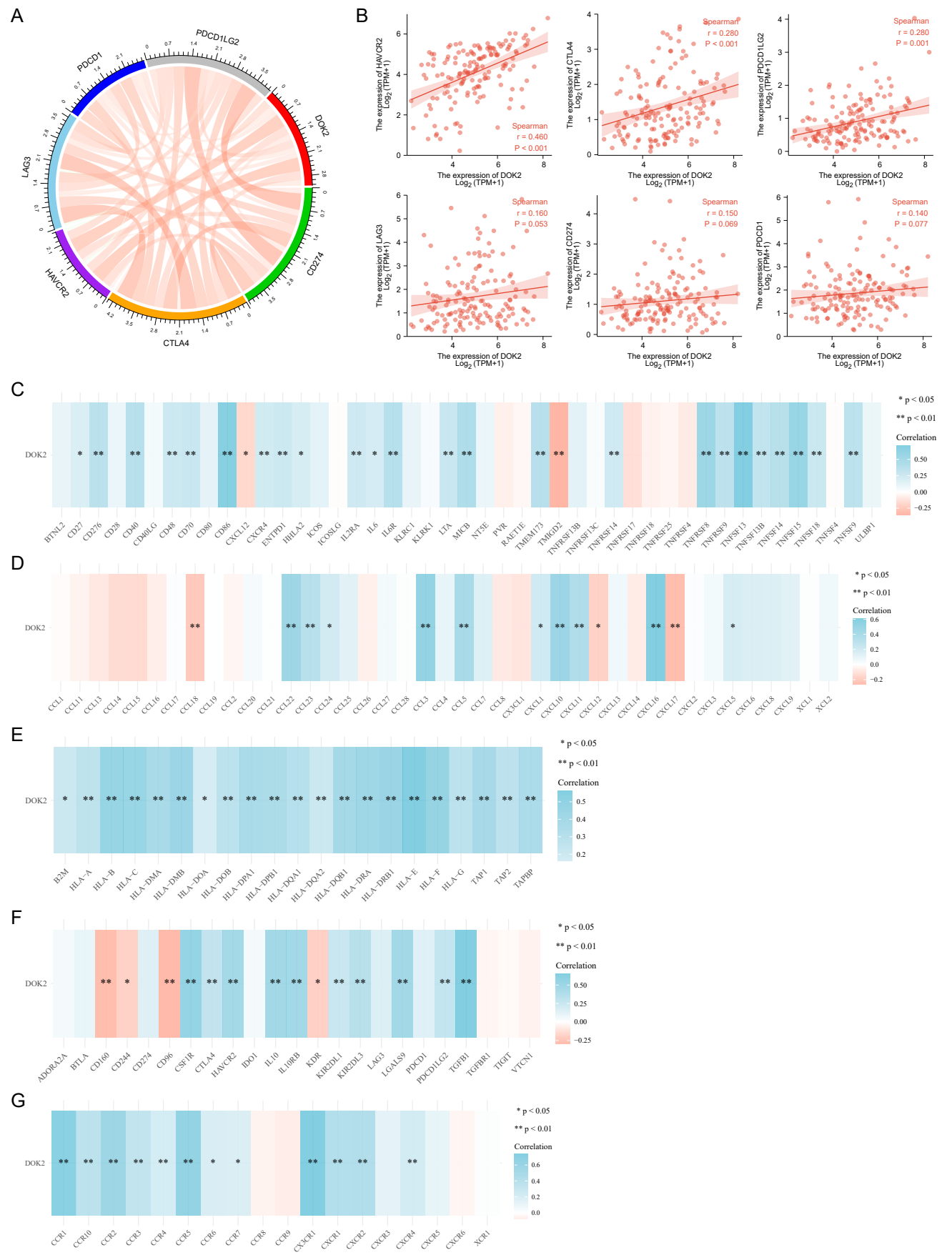

Supplement: Supplementary Figure S5 — Spearman correlation analysis between DOK2 and immune-related genes in the TCGA cohort. (A,B) Spearman correlations between DOK2 and immune checkpoint-related genes (HAVCR2, CTLA4, PDCD1LG2, LAG3, CD274, and PDCD1). (C) Spearman correlations between DOK2 and immune stimulators. (D) Spearman correlations between DOK2 and chemokines. (E) Spearman correlations between DOK2 and MHC molecules. (F) Spearman correlations between DOK2 and immune inhibitors. (G) Spearman correlations between DOK2 and chemokine receptors. [file Data_Sheet_5.pdf]

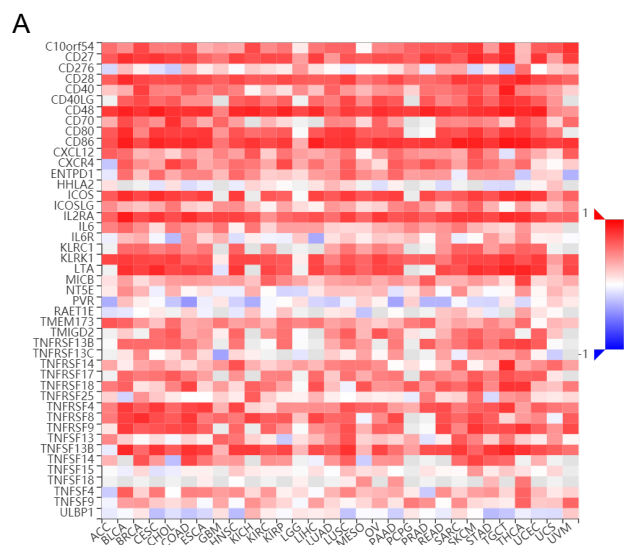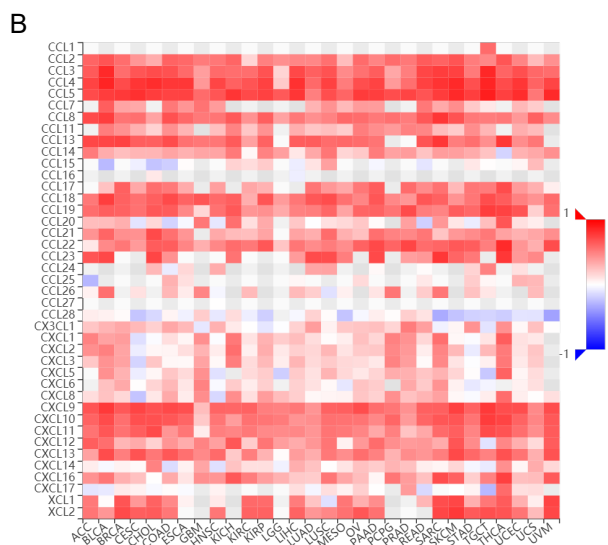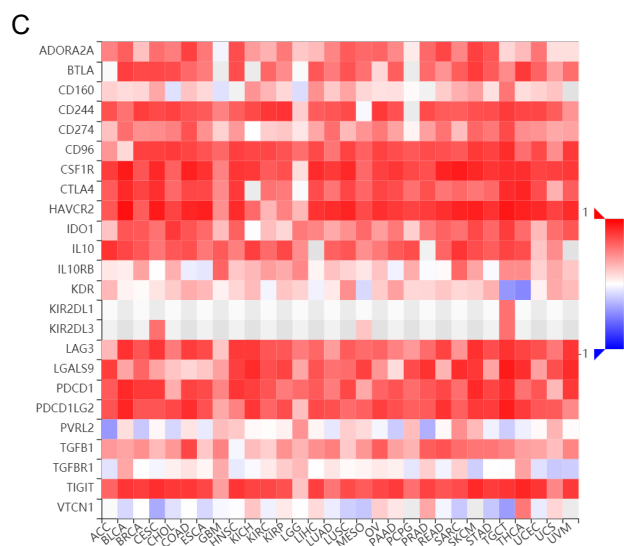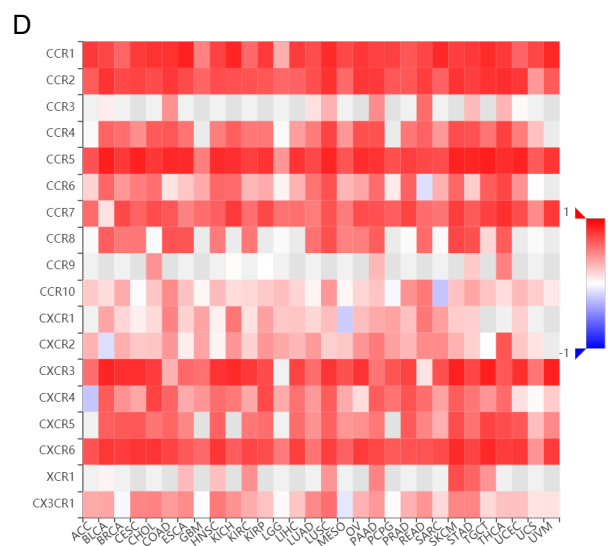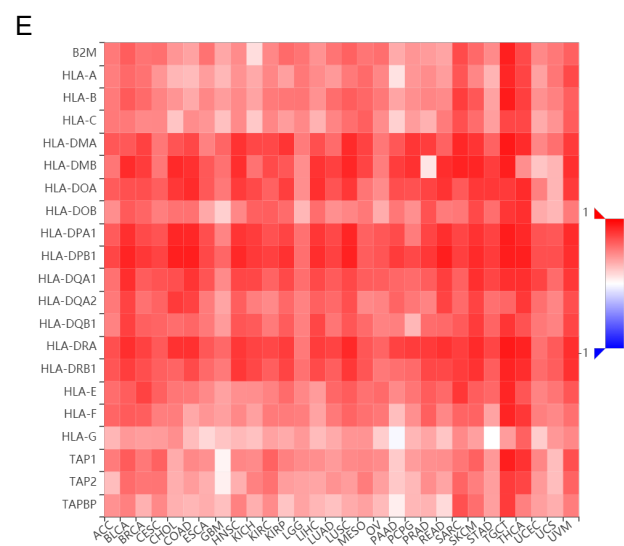

Supplement: Supplementary Figure S6 — Spearman correlations between DOK2 and immune-related genes in pan-cancer in TISIDB. (A) Spearman correlations between DOK2 and immune stimulators. (B) Spearman correlations between DOK2 and chemokines. (C) Spearman correlations between DOK2 and immune inhibitors. (D) Spearman correlations between DOK2 and chemokine receptors. (E) Spearman correlations between DOK2 and MHC molecules. [file Data_Sheet_6.pdf]
